# Molecular Biology: Challenges and Opportunities

**DOI:** 10.3390/cimb47020109

**Published:** 2025-02-09

**Authors:** Madhav Bhatia

**Affiliations:** Department of Pathology and Biomedical Science, University of Otago, 2 Riccarton Avenue, P.O. Box 4345, Christchurch 8140, New Zealand; madhav.bhatia@otago.ac.nz or madhav1245@gmail.com

“Latest Review Papers in Molecular Biology 2024”, a Special Issue of *Current Issues in Molecular Biology (CIMB)* that I have had the privilege of editing, called for articles on the challenges and opportunities in the field of molecular biology. Following an excellent response from researchers around the world, this Special Issue now boasts 29 published articles, which have all helped enhance our understanding of different aspects of molecular biology. The articles published in this Special Issue have already been viewed a total of 75,862 times at the time of writing of this Editorial (22 January 2025), and the number continues to rise daily.

All the articles in this Special Issue are of exceptionally high standards, and it has been my pleasure to read them. I would, therefore, encourage the readers of *CIMB* to read all the articles in this Special Issue, and I am sure you will enjoy reading them as I have. I would, however, like to highlight a few seminal review articles published in the Special Issue, which have dealt with complex ideas in an easy-to-read manner.

The first article [1] describes the use of collagen in cosmetic products. Collagen, a structural protein, is a frequently employed ingredient in several cosmetic products [2,3]. This review presented the types of collagen proteins, the factors affecting their structure, and their unusual role in the functions of the human body. The authors also summarize the type of cosmetic products where collagen is used, including cosmetics administered via injection, orally, as well as topically (creams, ointments, serums, cosmetic masks, etc.). This article elegantly deals with a complex subject, spanning from the basic chemistry of collagen to its practical applications in cosmetic products. In light of the increasing interest in this rapidly developing area, in the short time since it has been published, this article has already been viewed a total of 11,115 times.

The second article [4] is of outmost relevance to the present times, and it tackles the issue of micro- and nano-plastics and their impact on gut health. Micro- and nano-plastics are tiny particles derived from plastics, representing a significant health risk [5,6]. This review described recent research on the toxicological impact of micro- and nano-plastics on the gut, as observed in human cell lines and mammalian models. Available data suggest that these particles may result in adverse consequences, including epithelial toxicity, immune toxicity, and the disruption of the gut microbiota. This article concludes with directions for future research in this area, given the current limitations to our understanding of the subject. To date, this article has been viewed 4775 times.

The third article [7] describes the pharmacological effects of capsaicin. Capsaicin is the pungent compound found in chili peppers and, therefore, plays a significant role in the cuisine of several cultures around the world. Capsaicin acts through the TRPV1 (transient receptor potential cation channel subfamily V member 1) receptors. TRPV1 has been shown to play a key role in systemic inflammatory conditions such as sepsis [8,9,10]. The local application of capsaicin, however, produces an anti-inflammatory effect. Additionally, capsaicin has been reported to have anti-cancer and anti-microbial effects, with a long history of use in traditional medicine systems for several centuries. Though a major limitation in the therapeutic applications of capsaicin remains, which is related to its toxicity, especially in sensitive tissues. The research reviewed in this article suggests the potential of capsaicin in monotherapy or adjunct therapy for various pathologies. Further research is required in order to investigate capsaicin’s therapeutic properties that extend its usage beyond food. This article has been viewed 4064 times so far.

The fourth article [11] describes the application of multi-omics in biological systems. Biological systems are quite complex, and traditional approaches have limitations due to our incomplete understanding of the interactions within these systems. Every biological process may involve thousands of molecules, which traditional approaches are not capable of investigating. Biology-based multi-omics system approaches provide us with a global picture of complex biological systems, using approaches such as genomics, transcriptomics, proteomics, glycomics, glycoproteomics, metabolomics, and lipidomics analyses. Multi-omics approaches have thus far been used in the diagnosis for Alzheimer’s disease [12], the identification of osteoporosis biomarkers [13], and the diagnosis and therapy prostate cancer [14]. Investigating biological systems from a holistic viewpoint using multi-omics approaches, therefore, promises to significantly enhance our understanding of the diagnosis, treatment, and prevention of human diseases. This article describes the current state of knowledge and provides potential directions for the future of this very important and rapidly developing subject. This article has been viewed 4192 times so far.

Finally, I would like to highlight a review article that summarizes our current understanding of the positive effects of physical activity on insulin signaling [15]. Physical activity plays a vital role in maintaining metabolic health, particularly in addressing insulin resistance and the related disorders such as type 2 diabetes mellitus [16]. Research has shown that a strong association between physical activity levels and insulin sensitivity exists [17]. In this article, the author has described insulin signaling pathways, focusing on insulin signaling in skeletal muscles, the liver, and white adipose tissues. The article proceeds to elucidate the relationship between physical inactivity and islet cell insufficiency, which leads to the exacerbation of insulin resistance through various pathways including endoplasmic reticulum stress, mitochondrial dysfunction, oxidative stress, and inflammation. Physical activity, on the other hand, preserves and restores islet function, thus enhancing peripheral insulin sensitivity. Based on the literature on the molecular biology of insulin signaling, the author recommends that individuals be encouraged to adopt active lifestyles and engage in regular exercise. This may help them prevent and manage insulin resistance and related metabolic disorders, ultimately promoting overall health and well-being. Similarly to the other articles highlighted in this Editorial, this article deals with a complex subject but explains it clearly and comprehensibly. So far, this article has been viewed 4520 times.

In conclusion, this Special Issue represents a collection of review papers from a wide range of scientific fields. I am confident that the articles published herein will form the basis for more ground-breaking research in times to come. Encouraged by the response to this Special Issue in 2024—from the authors as well as readers—we are launching another Special Issue of *CIMB*, “Latest Review Papers in Molecular Biology 2025” (https://www.mdpi.com/journal/cimb/special_issues/5CV2UG4RK8, accessed on 22 January 2025), and I cordially invite you to contribute review articles pertaining to the challenges and opportunities in the discipline of molecular biology.
